# Beyond the Bench: Nurses Adapt to Changing Health Care Climate

**DOI:** 10.1289/ehp.113-a814

**Published:** 2005-12

**Authors:** Tanya Tillett

With increased emphasis being placed on the importance of environmental health comes the need for an expanded variety of environmental health care practitioners. Nursing is one of several professions that are augmenting and advancing the capabilities of their practitioners to meet this need. Now the Community Outreach and Education Program (COEP) of the University of New Mexico’s Center for Environmental Health Sciences has partnered with the New Mexico Environment Department to create an environmental health nursing internship as a component of its outreach program.

In cooperation with the university College of Nursing, the partners recruit nursing students who are interested in implementing environmental health care initiatives in the surrounding community. For four years, the program has taken nursing students beyond the traditional curriculum and shown them firsthand how interaction with environmental factors affects human health. The program also gives students valuable experience in taking measures to abate hazardous exposures in communities.

The interns are currently working on a number of projects that will affect different community members. They are helping to develop surveys and compiling data for a project investigating uranium exposure and subsequent kidney damage among the Navajo Nation, whose members live near and work in uranium mines. They are gathering and assembling materials for a community education and survey project on mercury in surface waters and other environmental health concerns among the Cheyenne River Sioux Tribe. They are helping COEP staff write and field-test an integrated environmental health curriculum on diabetes for middle school students. And in a fourth project, they are helping to develop a best-practices manual for applying farm waste fertilizer to croplands in a way that minimizes human exposure to aerosolized waste.

The local ties of some of the nursing interns have enhanced the program’s role as an effective community advocate. Intern Krystyn Yepa is a Native American from the Pueblo of Jemez who became interested in environmental health nursing because she wanted to understand some of the health effects in her tribe resulting from exposure to different pollutants in the environment. “Learning about the different environmental health problems that exist in New Mexico has given me the willpower to finish nursing school to ultimately achieve my goal of improving the health and lifestyles of my people by placing importance on the environment,” she says.

Yepa explains further, “As a Native American, I was raised to respect and appreciate the environment, and working at the COEP has only strengthened my values related to the environment.” She also credits the internship with teaching her important assessment tools that lay emphasis on the environment when completing a health history on patients, something a traditional nursing internship would not likely provide.

“The nursing interns have been an incredible asset to our COEP,” says staff member Stefani Hines. “It is a mutually beneficial situation on many levels—the student nurses gather valuable real-world experiences in environmental health; the nursing school has access to additional, unique placements for their students; we have additional support for projects, which helps make them better; and the communities we work with benefit from the students’ efforts as well.”

Dedicated, enthusiastic environmental health nursing interns will only continue to play an important role in advancing the COEP mission. One project currently in development will help community members get involved in city and county zoning processes, which will both encourage a healthy community mindset and minimize exposures to pollutants.

## Figures and Tables

**Figure:**
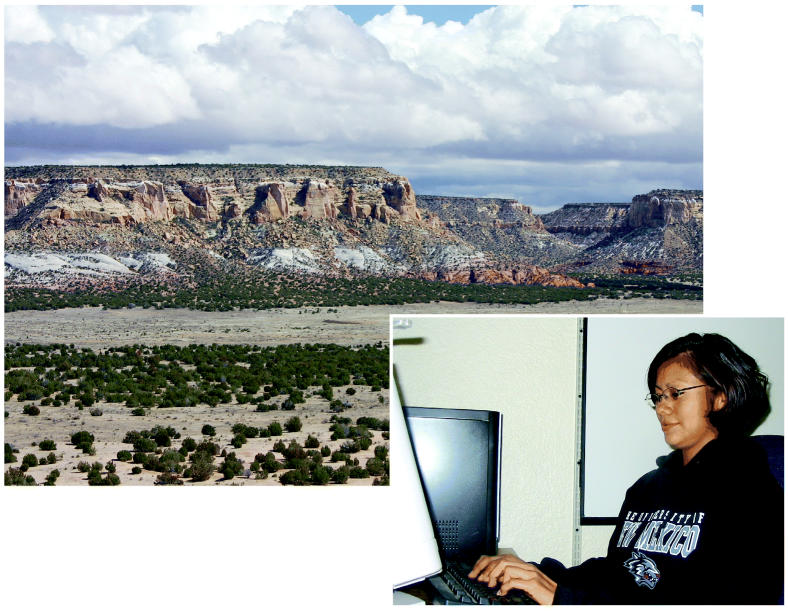
Effecting change in Southwest communities. Krystyn Yepa (right) is one of several nursing interns working with Navajo and Sioux communities to reduce exposures and increase knowledge through a program of the University of New Mexico Center for Environmental Health Sciences.

